# Identification of putative promoters in 48 eukaryotic genomes on the basis of DNA free energy

**DOI:** 10.1038/s41598-018-22129-8

**Published:** 2018-03-14

**Authors:** Venkata Rajesh Yella, Aditya Kumar, Manju Bansal

**Affiliations:** 1 Molecular Biophysics Unit, Indian Institute of Science, Bangalore, Karnataka 560012 India; 2 Department of Biotechnology, Koneru Lakshmaiah Education Foundation, Vaddeswaram, Guntur, Andhra Pradesh 522502 India; 3 Present Address: Department of Molecular Biology and Biotechnology, Tezpur University, Tezpur, Napaam, Assam 784028 India

## Abstract

Transcription is an intricate mechanism and is orchestrated at the promoter region. The cognate motifs in the promoters are observed in only a subset of total genes across different domains of life. Hence, sequence-motif based promoter prediction may not be a holistic approach for whole genomes. Conversely, the DNA structural property, duplex stability is a characteristic of promoters and can be used to delineate them from other genomic sequences. In this study, we have used a DNA duplex stability based algorithm ‘PromPredict’ for promoter prediction in a broad range of eukaryotes, representing various species of yeast, worm, fly, fish, and mammal. Efficiency of the software has been tested in promoter regions of 48 eukaryotic systems. PromPredict achieves recall values, which range from 68 to 92% in various eukaryotes. PromPredict performs well in mammals, although their core promoter regions are GC rich. ‘PromPredict’ has also been tested for its ability to predict promoter regions for various transcript classes (coding and non-coding), TATA-containing and TATA-less promoters as well as on promoter sequences belonging to different gene expression variability categories. The results support the idea that differential DNA duplex stability is a potential predictor of promoter regions in various genomes.

## Introduction

Genetic transcription program is initiated in a segment of DNA referred to as ‘promoter’ which serves as a platform for the assembly of pre-initiation complex to specify the transcription start sites (TSSs)^1^. In eukaryotes, promoter architecture is quite complex and diverse. Eukaryotic promoters are broadly classified as core promoters, proximal promoters and distal promoters. Core promoter regions are characterized by the presence of cognate sequence motifs such as Initiator (Inr), TATA-box and downstream promoter element (DPE) positioned at distinct locations relative to TSS, as well as non-canonical elements such as “ATG deserts” and “CpG islands” in mammals^2–4^. Proximal promoter regions are the sequences spanning region 500 base pairs relative to the TSS and contain certain sequence elements, which include the CAAT box, the GC box and *cis*-regulatory modules^5^. Distal promoter elements encompass enhancers, insulators and silencers. Based on the strategies of transcription initiation, core promoters can be broadly classified as focused core promoters or dispersed core promoters^6,7^. The promoter region for the genes with dispersed transcription initiation can occur several 1000s of nucleotides upstream of the gene body. Further, from the studies on genome-wide nucleosome density maps of different eukaryotes such as *Saccharomyces cerevisiae*, *Schizosaccharomyces pombe*, *Drosophila melanogaster* and humans, it has been revealed that well-positioned nucleosomes usually occupy the sequences downstream of TSS, and the core promoter regions are devoid of the nucleosome^8^. Recent analyses of eukaryotic promoters indicates that though promoters differ in their sequence context (most of them lack consensus motif) and GC composition (lower eukaryotes are AT rich while mammals are GC rich), some properties such as nucleosome-free region and epigenetic features around transcription start sites are quite common^9,10^. The complexity of eukaryotic promoter architecture is further revealed by the discovery of alternative^6,7^ and bidirectional promoters^11,12^. In addition, recent experimental studies showed that the transcription landscape in eukaryotes is quite pervasive in nature, with a high proportion of transcripts originating from intergenic regions^12,13^. With this avalanche of genomic sequence data, it is important to characterize and predict promoter sequences in order to fully understand the process of transcription initiation.

The delineation of promoters is also essential for complete annotation of genomes and a better understanding of genome’s regulatory networks and their architecture^14^. Experimental methods for locating promoter elements include techniques such as 5′-tag-based methods which characterize promoters and transcription initiation events, small RNA sequencing, as well as methods which capture DNA-bound proteins, including RNAPII, transcription factors and histone modifications^9^. They provide a snapshot of all transcribed regions or DNA-protein interactions in the genome for specific experimental conditions^7,10^. Experimental techniques are costly, labour-intensive and time-consuming. Alternatively, computational methods provide rapid and relatively inexpensive ways for promoter identification. These methods are mostly based on the basic premise that promoter sequences have unique statistical features when compared to other genomic sequences^10^. These approaches use DNA sequence feature information such as biological signals of core promoter elements (Inr, TATA-box, and CpG islands), statistical properties of k-mer composition and DNA secondary structural features. Structural features are found to be more informative and universal as compared to sequence or base-compositional features. DNA structural features such as flexibility/bendability, curvature, base stacking and duplex stability have been applied to characterize promoter regions^15–19^. They are better predictors, as the structural features are comparatively conserved, more informative and widely applicable across genomes. Recent reports suggest that structural properties are linked to variability of gene expression^15,20,21^ and help in understanding different promoter classes^22^. Recent findings reveal that, DNA shape features can be useful in understanding, and characterizing transcription factor binding sites, origins of replications and other genomic regions^23–26^. The majority of the structure-based algorithms have been designed with the aim of annotating promoter regions specifically in humans, while a few algorithms such as “PromPredict”^17,27,28^ and “EP3”^16^ are applicable across a variety of genomes. It has also been reported that among all structural features examined, energy-based features such as base stacking or DNA duplex stability are better predictors^16^.

DNA duplex stability, a secondary structural feature can be expressed in terms of short range nearest-neighbour interactions, inter base hydrogen bonds and stacking interactions, which are explicitly dependent on identity and orientation of flanking base pairs^14^. DNA duplex stability computation has been applied in techniques such as PCR, anti-gene targeting, and for understanding replication, repair, and transcription^29,30^. PromPredict algorithm encodes the dinucleotide free energy information obtained from studies of melting temperatures of oligonucleotides to compute the average free energy of a particular sequence, as an indicator of DNA duplex stability. The strategy behind using DNA duplex stability is that promoter regions should be less stable than flanking regions to facilitate melting at the time of transcription initiation. Although PromPredict was developed for bacterial promoter prediction^17,27^, it also works very well for plants^28,31^, but its efficiency has not been tested on fungal and metazoan promoter sequences. In comparison, EP3, which also uses base-stacking energy, has been applied to annotate promoter regions in several eukaryotes ranging from protists to mammals^16^. In the present study, we report the validation of ‘PromPredict’ in identification of the promoter sequences of 48 eukaryotic systems.

The key elements of this *in silico* study are:

(i) Understanding the variation of DNA duplex stability eukaryotes,
(ii) Evaluating performance of PromPredict across various eukaryotes,
(iii) Whole genome promoter Prediction for different classes of transcripts in *S*. *cerevisiae*. An analysis of predictions for various gene expression variability classes and TATA-containing and TATA-less promoters in *S*. *cerevisiae*.

## Results and Discussion

The efficiency of in-house software PromPredict, a promoter prediction algorithm that demarcates putative promoter regions based on DNA duplex stability has been studied extensively in archaea, bacteria^17^ and plants (Rice and Arabidopsis)^28^. Current study deals with the promoter prediction analysis in the genome sequences of 48 different eukaryotes with translation start site (TLS) data, along with genome sequences of *S*. *cerevisiae*, *C*. *elegans*, *D*. *melanogaster*, zebrafish, mouse and human whose transcription start site (TSS) data is available. The true positive region for predictions for all systems is considered to be −500 to +100 relative to TLS/TSS in all cases except when comparing PromPredict with EP3. Initially DNA duplex profiles in these systems have been compared qualitatively.

### Eukaryotic promoters show distinct low stability region

**Figure 1:**
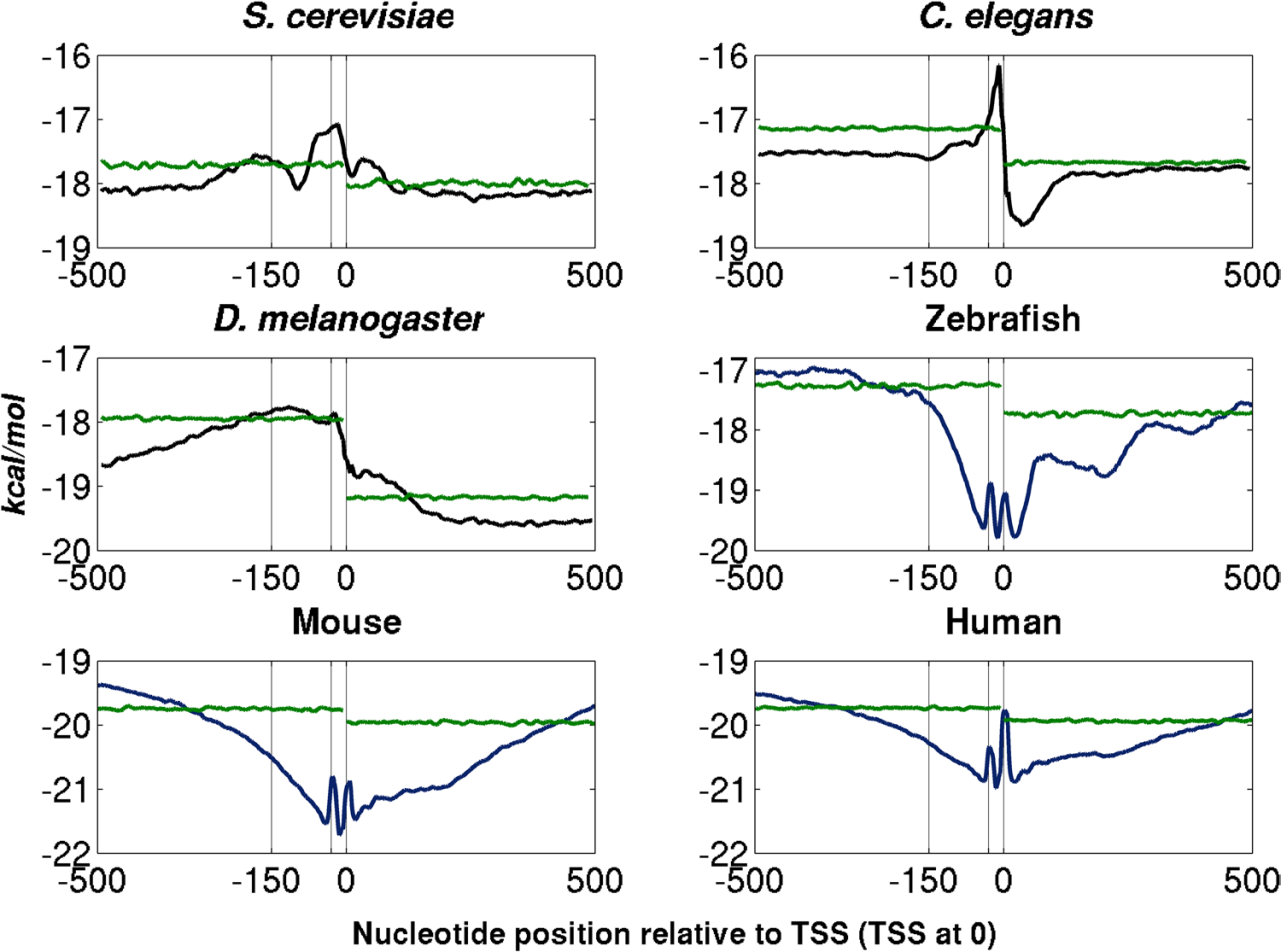
DNA duplex stability profiles of *S*. *cerevisiae*, *C*. *elegans*, *D*. *melanogaster*, zebrafish, mouse, and human. The two vertical lines correspond to −150 and −30 position with respect to TSS. Promoter regions in yeast and invertebrates are less stable. Low free energy peaks are observed at −19, −11 and −114 for *S*. *cerevisiae*, *C*. *elegans* and *D*. *melanogaster* respectively. Vertebrate core promoters have two narrow low stability peaks near the vicinity of TSS. Two narrow peak regions are observed at −27, +2 for zebrafish, −29, +6 for mouse and −30 and +1 for human. The stability (average free energy) profiles of genomic promoter sequences are plotted in blue colour while those for shuffled promoter sequences are shown in green.

The average DNA duplex stability profiles of promoter regions of six systems with mapped TSS data are presented in Figure 1 (data can be found in additional file). Promoter regions (−500 to +100 relative to TSS) in *S*. *cerevisiae*, *C*. *elegans* and *D*. *melanogaster* show low stability compared to their downstream regions, with narrow less stable regions being observed at −19, −11 and −114 (split peak at −25) for *S*. *cerevisiae*, *C*. *elegans*, and *D*. *melanogaster* respectively. In the case of vertebrates, zebrafish, mouse and human, core promoter regions are GC-rich but have two narrow low free energy peaks in the vicinity of TSS. Two narrow peaks are observed at −27 and +2 for zebrafish, −29 and +6 for mouse and −30 and +1 for human, which may correspond to the locations of TATA-box and INR (initiator) elements respectively (additional file). Similar low stability regions have been observed in different prokaryotic systems and plants^21,28,32^. The DNA duplex stability profiles of yeast and invertebrates are similar to prokaryotic promoters. In the case of a majority of prokaryotes, irrespective of genome GC content, promoter regions are less stable (or AT-rich) compared to downstream region, while in vertebrate promoters they are GC-rich, with several sequence and structure elements such as CpG islands, G-tracts, and G-quadruplex motifs being present in the vicinity of TSS^21^. GC contents of six eukaryotic systems in different regions of promoters, −500 to +500, −500 to −100, −100 to −1, +1 to +100 and +100 to +500 are given in (Supplementary Figure 1). The core promoter regions (−100 to −1) in yeast and invertebrates display less GC content compared to other regions as well as genomic GC while in mammals all regions prefer high GC content compared to genome GC.

**Figure 2:**
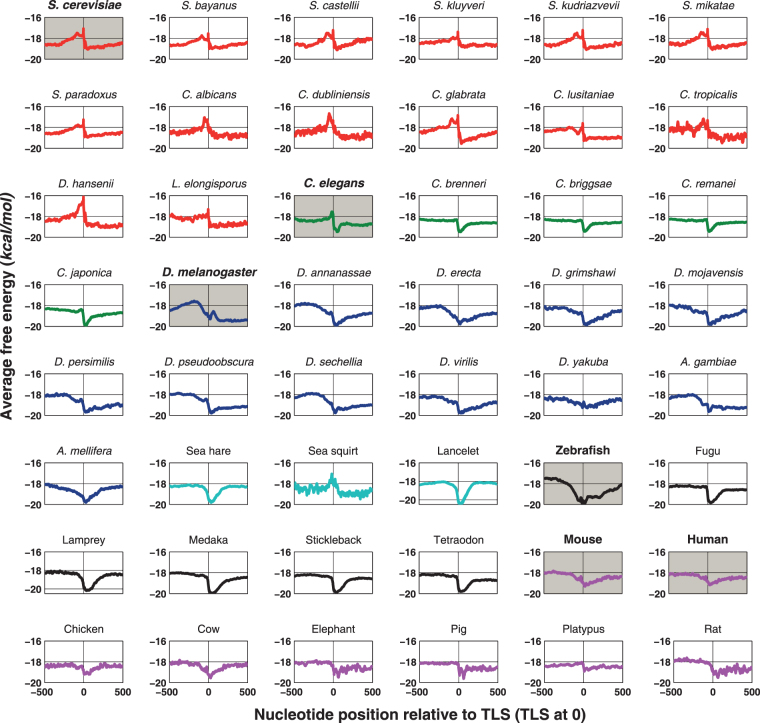
Average free energy profiles of promoters regions in different eukaryotes belonging to fungi, invertebrates (worm and fly) and vertebrates. The promoter sequences (−500 to +500 relative to translation start site (TLS)) are downloaded from SGD, CGD, and UCSC genome browser. The promoter sequences with GC percentage range 40–45 have been plotted. The red, green, blue, cyan, black and pink color plots represent fungi, worm, fly, marine invertebrate, fish and mammal including bird respectively. The model systems *S*. *cerevisiae*, *C*. *elegans*, *D*. *melanogaster*, zebrafish, mouse, and human are highlighted with gray background. The trends in AFE profiles are unique to closely related classes.

The DNA duplex stability profiles show a similar trend in promoter regions of closely related species (Fig. 2, additional file). To compare this feature in different eukaryotes, the promoter sequences (−500 to +500 relative to TLS) with (40–45) % GC composition are chosen and average free energy is plotted. All fungal species display free energy profiles similar to that of *S*. *cerevisiae* in the vicinity of TLS, while fish, sea hare and lancelot show a dip at immediate downstream region of TLS. An extensive study by Kumari S. and Ware D. reported the characteristic signatures of free energy profiles in eight plant species^31^. Further, they showed variations in the free energy profiles of monocot and dicot plants. Our results suggest that unique patterns of DNA duplex free energy or stability are conserved among closely related eukaryotes. It is interesting to see the validation of PromPredict in two different types of promoter classes; AT-rich in yeast and invertebrates and GC-rich in vertebrate promoters.

### Promoter prediction using PromPredict

Promoter prediction in 48 eukaryotic systems with TLS data (14 species of yeast, five species of worm, 12 species of fly, three marine invertebrates, six fish species, seven mammals and chicken) and six eukaryotic systems, *S*. *cerevisiae*, *C*. *elegans* and *D*. *melanogaster*, zebrafish, mouse and human with mapped TSS data has been carried out using PromPredict. The program discriminates putative promoters from other genomic sequences using relative differences in average free energy and cut-offs based on GC content of the sequences (methods). PromPredict gives predictions in 5′ to 3′ direction and can be applied to the whole genome as well as shorter fragments (not less than 1000 nt). Promoter prediction has been carried out using two strategies; (i) 1001mer or 2001mer sequences and (ii) on whole genome. The analysis of shorter fragments has been performed for 48 systems with TLS data and six systems with TSS data. While the second approach, *viz* whole genome annotation, is utilized for *S*. *cerevisiae*. In all cases, the true positive region is considered to be 600 nt of length, extending from −500 nt upstream to +100 nt downstream, relative to TSS or TLS. Least stable positions (lsp) are considered to evaluate the predictions. Since this kind of analysis gives only true positives, only recall values are derived to assess the performance of PromPredict.

### Performance of PromPredict on 48 systems with TLS data

**Figure 3:**
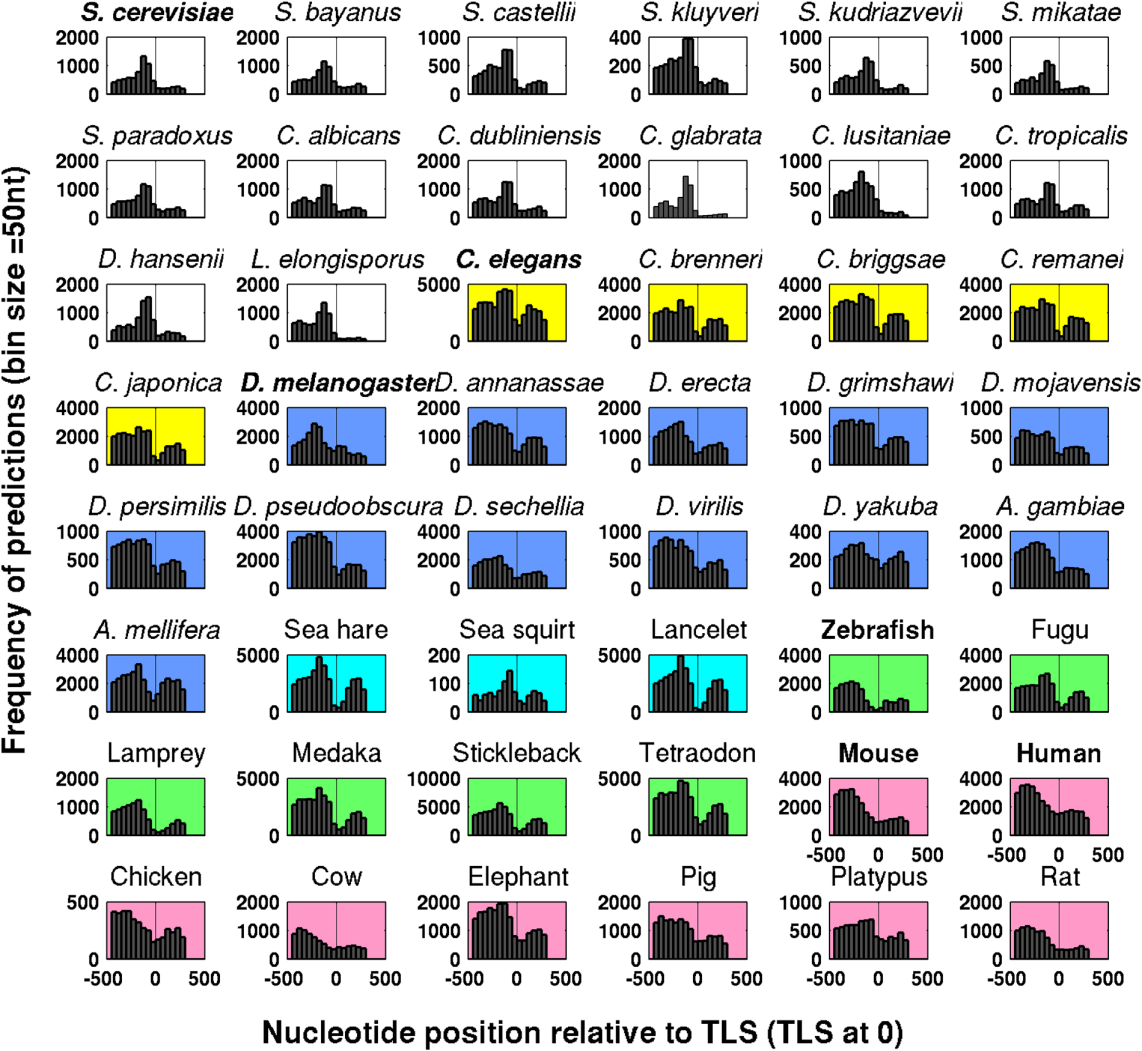
Positional distribution of predictions in promoter regions of different eukaryotes. The plot shows the distance of predictions from TLS in 50 nt bins for 48 different eukaryotes representing 14 species of yeast, five species of worm, 12 species of fly, three marine invertebrates, six fish species, seven mammals, and chicken. Color codes for the background of subplots (white for fungi, yellow for the worm, blue for fly, cyan for marine invertebrates, green for fishes and pink for mammals and bird) have been used to differentiate between different domains of life.

The 48 eukaryotic systems represent species closely related to the six systems with TSS data, along with distantly related systems such as marine invertebrates. They constitute 14 species of yeast, five species of worm, and 12 species of fly, three marine invertebrates, six fish species, seven mammals and chicken (Table 1). The genomic size of these eukaryotes ranges from 10.8 mb (for *S*. *mikatae*) to 3197 mb (for elephant) while genomic GC varies from 33% (for *C*. *dubliniensis*) to 47% (for lamprey). PromPredict has been tested on all these systems considering −500 to +100 region relative to TLS as true positive (TP) region and the recall values are calculated to evaluate the performance. Figure 3 shows the positional distribution of predictions (lsp) in −500 to +500 regions in different eukaryotic promoters. Since PromPredict gives predictions by considering 250 nucleotide (nt) sliding window (two 100 nt windows separated by 50 nt region) predictions are only obtained up to 750 nt position in a 1000 nt sequence. In all studied systems the frequency of lsp in upstream 50 nt bins is consistently higher as compared to downstream regions. In 14 species of yeast, the lsp signal is observed immediately upstream of TLS (approximately −150 to −50). The predictions in worm and fly species are spread over the upstream region, extending till −500 and possibly beyond. Mammals such as human, mouse, cow and pig show higher predictions in far upstream regions, beyond −250, from the TLS. Overall the trends in the distribution of predictions are similar to that of the related systems with TSS position as reference.

**Table 1:** Promoter prediction in 48 different eukaryotes.

	Genome size in Mb	Genome GC %	Promoter sequences	Total predictions	TP predictions	TP genes	Recall
**Yeast**							
*S*. *cerevisiae*	12.5	38.4	6642	8113	6636	5386	81.1
*S*. *bayanus*	11.9	40.3	7216	8161	6415	5346	74.1
*S*. *castellii*	11.4	37	4655	5745	4528	3703	79.5
*S*. *kluyveri*	11	41.7	2932	2991	2433	2059	70.2
*S*. *kudriazvevii*	11.2	39.9	3736	4157	3431	2863	76.6
*S*. *mikatae*	10.8	38.2	3064	3773	3054	2465	80.5
*S*. *paradoxus*	11.9	38.6	7373	8578	6807	5608	76.1
*C*. *albicans*	14.5	33.7	5852	8628	6764	5063	86.5
*C*. *dubliniensis*	14.6	33.2	5933	8988	7114	5255	88.6
*C*. *glabrata*	12.1	38.5	5149	6913	5913	4575	88.9
*C*. *lusitaniae*	12.1	44.5	5797	5621	4867	4051	69.9
*C*. *tropicalis*	15.3	33.5	6119	8855	6714	5112	83.5
*D*. *hansenii*	11.5	35.4	6102	9100	7459	5592	91.6
*L*. *elongisporus*	15.5	37	5657	8337	6933	5076	89.7
**Worm**							
*C*. *elegans*	98.3	35.4	32481	49575	34895	26474	81.5
*C*. *brenneri*	190.4	39.7	24989	28345	19993	16714	66.9
*C*. *briggsae*	108.4	37.7	31342	36290	25460	21187	67.6
*C*. *remanei*	145.4	38.5	26174	30728	21571	17874	68.3
*C*. *japonica*	166.3	39.9	22173	28033	19710	16061	72.4
**Fly**							
*D*. *melanogaster*	143.7	42.1	17283	24152	18720	14536	84.1
*D*. *annanassae*	231	42.5	13677	18831	12513	10127	74.0
*D*. *erecta*	152.7	42.6	12395	15917	10887	8996	72.6
*D*. *grimshawi*	200.5	38.8	7861	10009	6822	5498	69.9
*D*. *mojavensis*	193.8	40.2	5329	7172	4982	3885	72.9
*D*. *persimilis*	188.4	45.2	7598	10487	7452	5812	76.5
*D*. *pseudoobscura*	152.7	45.3	31482	45081	32400	25117	79.8
*D*. *sechellia*	166.6	42.5	19059	25110	17308	13974	73.3
*D*. *virilis*	206	40.7	7920	10433	7238	5673	71.6
*D*. *yakuba*	165.7	42.4	2857	3951	2619	2105	73.7
*A*. *gambiae*	265	44.5	13901	17893	13044	10487	75.4
*A*. *mellifera*	250.3	34.1	21146	36526	23533	17690	83.7
**Marine invertebrates**							
Sea hare	927.3	42	33340	41949	28465	23737	71.2
Sea squirt	116.7	36.1	729	1073	748	591	81.1
Lancelet	521.9	41.8	30538	41175	27789	23402	76.6
**Fishes**							
Zebrafish	1371.7	36.7	14404	20036	13780	11329	78.7
Fugu	391.5	45.8	18679	25638	17793	14685	78.6
Lamprey	885.5	46.8	8724	11355	8087	6839	78.4
Medaka	869.8	42.3	29255	40229	27999	23106	79.0
Stickleback	446.6	42	38971	54946	37856	30987	79.5
Tetraodon	342.4	46.3	34618	50425	34481	27838	80.4
**Mammals and bird**							
Mouse	2803.6	41.9	23878	34040	23118	18661	78.2
Human	2851.4	40.9	26800	41102	27229	21419	79.9
Chicken	1046.9	41.9	3707	5292	3353	2753	74.3
Cow	2983.3	42.3	7514	11110	7338	5849	77.8
Elephant	3196.7	40.9	16095	22427	15638	12580	78.2
Pig	2808.5	42.5	11953	18064	12403	9736	81.5
Platypus	1995.6	45.7	6166	8336	5969	4735	76.8
Rat	2616.4	42.4	8567	11987	8498	6817	79.6

Promoter prediction in 14 species of yeast, five species of worm, 12 species of fly, three marine invertebrates, six fish species, seven mammals and chicken are carried out using PromPredict algorithm. The promoter regions, −500 to +500 relative to TLS (TLS at 0) are considered for this analysis and are retrieved from SGD, CGD, and UCSC genome browsers. The −500 to +100 relative to TLS is considered as true positive region.

**Figure 4:**
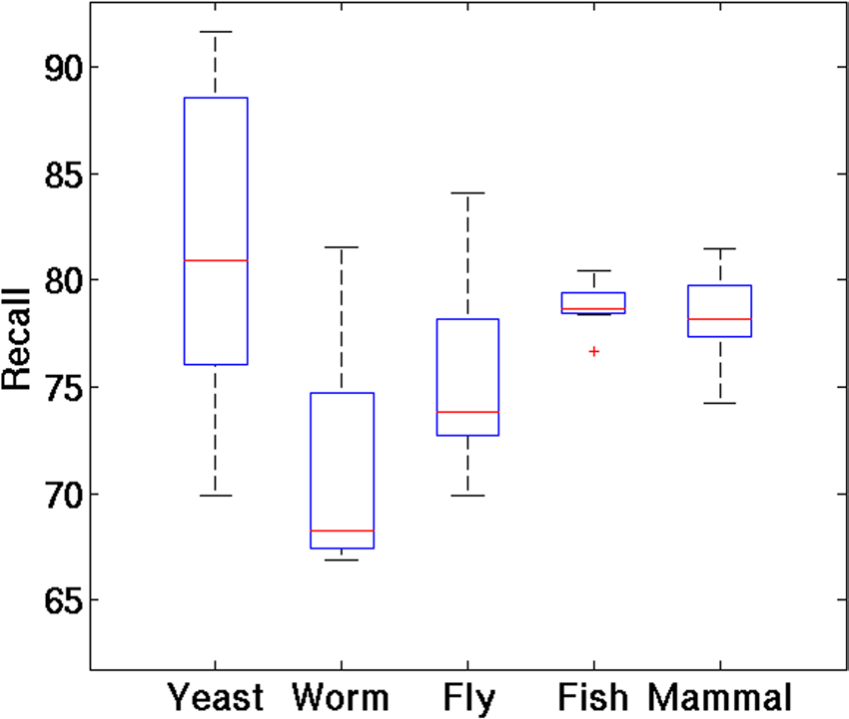
Performance of PromPredict in different eukaryotes. The box plot shows the recall in 14 species of yeast, five species of worm, and 12 species of fly, three marine invertebrates, six fish species, seven mammals and chicken. The −500 to +100 region relative to TLS is considered as true positive region in this study.

The percentage recall values for the 48 systems range from 67.6 to 91.6 for *C*. *briggsae* (worm) and *D*. *hansenii* respectively (Table 1, Fig. 4). In yeast, the recall values span the widest range from 70.4 to 91.6. The 14 yeast species show similar free energy profiles, but they differ considerably in their genomic GC content. The recall values for yeast species with high GC content such as *S*. *bayanus*, *S*. *kluyveri* and *C*. *lusitaniae* have lower recall values whereas genome with less GC content such as *C*. *dubliniensis*, *C*. *albicans* and *D*. *hansenii* show higher recall values. The correlation between genome GC and recall is observed to be −0.79 (*p* = *0*.*0007*) (Supplementary Figure 2). The reason for this kind of relationship in yeasts may be due to the compactness of the genomes; the GC content of genome is directly reflected in GC content of promoter regions. There is no such relationship observed for genome GC and recall values for invertebrates or vertebrates. However, it should be noted that PromPredict performs better in less GC-rich genomes compared to high GC-rich genomes. The performance of PromPredict is weak in worm species except for *C*. *elegans* as they do not have a prominent free energy peak in the vicinity of TLS (Fig. 2). In mammals, PromPredict shows lower prediction accuracy when compared to invertebrates. Invertebrates and mammals have unique structural motifs (A-tracts in invertebrates and G-quadruplexes in mammals). Supplementary Figures 3 and 4 show the distribution of A-tracts and G-quadruplexes in 48 eukaryotes. The PromPredict analysis is tested further in six eukaryotes with experimentally validated TSS data.

### Performance of PromPredict on six systems with TSS data

The six systems considered are *S*. *cerevisiae*, *C*. *elegans*, *D*. *melanogaster*, zebrafish, mouse, and human, as representatives of different domains of life, ranging from single-celled yeast to complex mammalian systems. The whole genome GC content varies from ~35% (in *C*. *elegans*) to ~42% (*D*. *melanogaster* and mouse). Prediction calculations are restricted from −500 to +500 regions for *S*. *cerevisiae*, *C*. *elegans*, and *D*. *melanogaster* while from −1000 to +1000 regions are considered in the case of vertebrates. The positional distribution of least stable position in 50 nt binned windows in six systems shows that the location of prediction varies in yeast, invertebrates, and vertebrates (Supplementary Figure 5). In *S*. *cerevisiae*, *C*. *elegans* and *D*. *melanogaster* majority of the predictions are located in the upstream vicinity of TSS (approximately −1 to −150 region) whereas in mammals, maximum number of predictions is observed beyond −300 region relative to TSS. Percentage recall values for the six systems are 85.1, 87.7, 91.9, 80.4, 67.2 and 71.3 for *S*. *cerevisiae*, *C*. *elegans*, *D*. *melanogaster*, zebrafish, mouse, and human respectively (Table 2). Higher recall values are observed for lower eukaryotes as expected, but surprisingly good recall values are also obtained for mouse and human.

**Table 2:** Promoter predictions in six systems using “PromPredict” algorithm.

	Promoter sequences	Genome GC	Total predictions (−500 to +500 w.r.t TSS at ‘0’)	TP (−500 to 100)	GC% in TP region	TP genes	Recall
*S*. *cerevisiae*	4912	38.3	11227	6312	37.6	4182	85.1
*C*. *elegans*	18457	35.4	28823	23063	35.1	16178	87.7
*D*. *melanogaster*	12898	42.1	18332	16232	39.8	11856	91.9
Zebrafish	5366	36.1	8220	5464	37.6	4313	80.4
Mouse	17451	41.9	22017	14281	54.6	11721	67.2
Human	29456	40.9	43043	27138	53.1	21009	71.3

Predictions in −500 to +500 regions w.r.t TSS of *S*. *cerevisiae*, *C*. *elegans* and *D*. *Melanogaster*, zebrafish, mouse and human have been carried out. PromPredict performs better in the case of yeast and invertebrates as compared to mammals. The −500 to +100 region relative to TSS is considered as true positive region.

To check the distribution of predictions, different TP classes are defined with each class representing the promoter sequences having a prediction within 50 nt bins (shown in Supplementary Figure 5) spanning the regions −1 to −50, −51 to −100, −101 to −150, *etc*. The average free energy profiles of different TP classes (Supplementary Figure 6) indicate that each class has a broad low stability region corresponding to the bin position, in all systems. These distinct stability profiles in the promoter regions may explain the abundance of *cis*-regulatory elements in the proximal as well as distal regions of TSS^28^. The results presented here suggested that PromPredict can be potential algorithm for promoter prediction in eukaryotes. Further, the performance of PromPredict has been compared with EP3.

### Comparison of PromPredict performance with EP3

**Figure 5:**
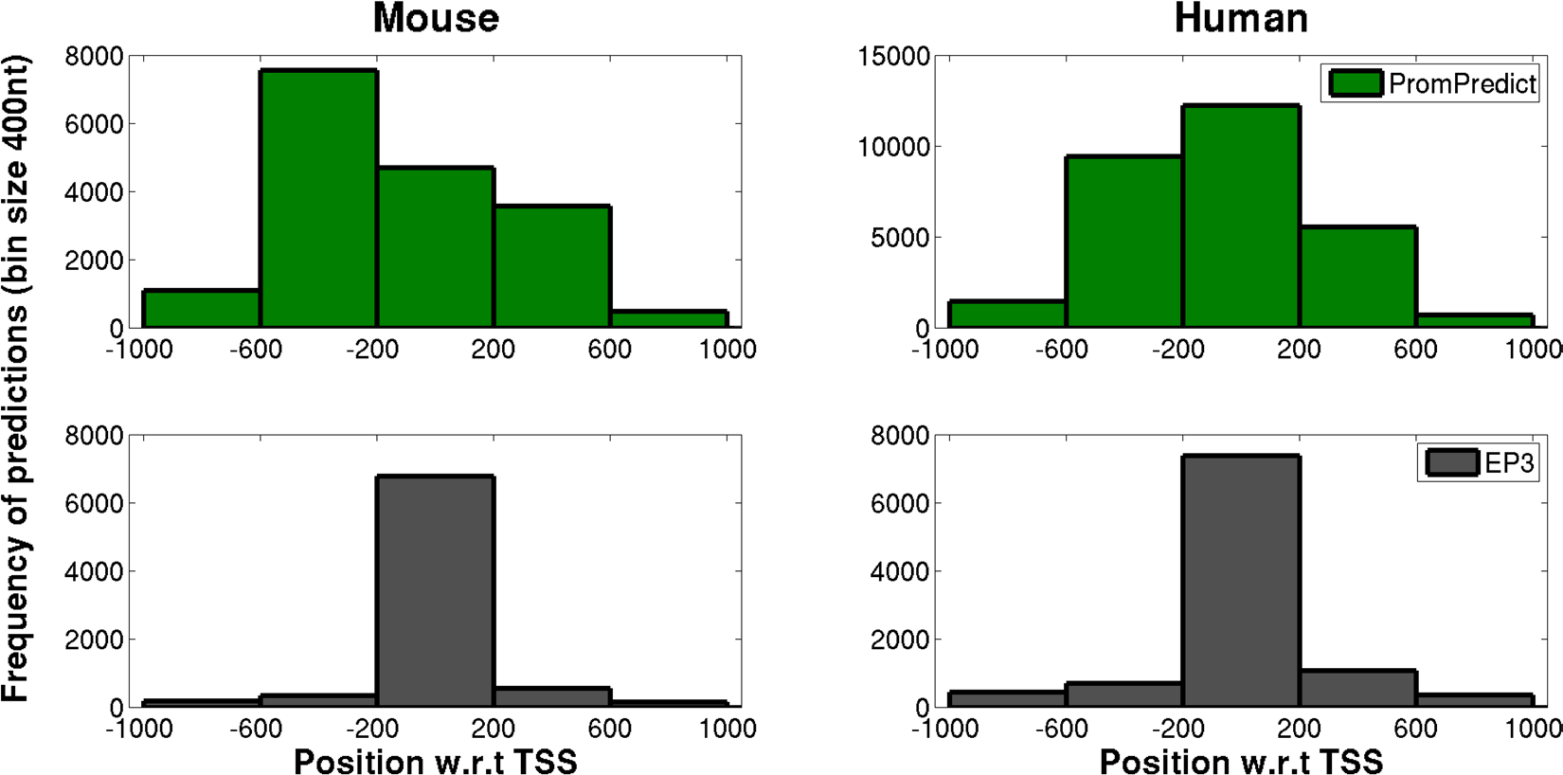
Distribution of ‘TP’ promoter predictions by PromPredict and EP3. Predictions in −1000 to +1000 region relative to TSS for both mouse and human have been considered. PromPredict uses a 250 nt sliding window with the midpoint of a 100 nt window being considered as a prediction, if it satisfies GC based cut-offs, while EP3 prediction is given in a 400 nt non-overlapping window and defines the window as a true or false prediction based on genome-specific thresholds. In case more than one ‘TP’ prediction is obtained for a gene, then the position closest to TSS has been considered. Hence, the total number of TPs plotted corresponds to ‘TP genes’.

The performance of PromPredict has been compared with the other free energy based promoter predictor EP3^16^ which has been applied primarily to predict mouse and human promoters (in the −1000 to +1000 nt regions, where TSS is centred at ‘0’ position). Hence the True Positive (TP) region for both predictors is considered as spanning −1000 to +1000 region. The EP3 program uses base-stacking property to distinguish promoter regions from non-promoter sequences. For a given sequence of DNA, it calculates inverted base-stacking energy values over non-overlapping window of 400 base pairs size and calls a region as a promoter when the structural feature value crosses a prescribed threshold score, which is genome specific. So for EP3 every 400 nt window has a prediction, but with true or false prediction value assigned to it, while PromPredict gives only predicted promoter regions in a continuously sliding window. PromPredict provides majority of the predictions in −600 to +200 regions with respect to TSS in both mouse and human, whereas EP3 gives predictions in −200 to +200 regions. The distribution of closest TP predictions shown in Figure 5 suggests that both EP3 and PromPredict are good predictors of promoters. However, as seen from Table 3, the recall values for EP3 are very low as compared to PromPredict for both human and mouse. PromPredict gives recall values of 99% for both mouse and human whereas EP3 gives 46% and 36% recall for mouse and human respectively which are comparable to whole genome prediction values (recall ~41% with CAGE dataset) reported in their study^16^. The results suggest that PromPredict is a better predictor than EP3, even though latter has been trained on the human genome. Since the entire 2001 nt region spanning TSS is considered as ‘TP’ region, no false positives have been identified and hence ‘Precision’ values have not been obtained in this study. PromPredict algorithm was further tested in various classes of transcripts, promoter types and gene expression datasets.

**Table 3:** Comparison between performance of PromPredict and EP3.

		Promoter sequences	Total predictions in −1000 to +1000nt region w.r.t TSS at ‘0’	TP	TP genes	Recall
PromPredict	Mouse	17451	50834	50834	17301	99.1
	Human	29456	90622	90622	29246	99.3
EP3	Mouse	17451	87255	13336	8052	46.1
	Human	29456	147279	18810	10522	35.7

Prediction for −1000 to +1000 region w.r.t TSS for both human and mouse are used for prediction. The whole region −1000 to +1000 region has been used as a truepositiveregion^16^ to calculate recall values. PromPredict gives far better recall value when compared to EP3.

### Whole genome promoter prediction in *S*. *cerevisiae*

*S*. *cerevisiae* is an important model organism for eukaryotes, its genome being compact with genes representing ~72% of the entire sequence. Promoter prediction has been carried out for all 16 chromosomes in both forward and reverse strands separately. The predictions are mapped to the −500 to +100 region relative to TSS of each gene. If the least stable position (lsp) of the predicted promoter region lies within the 500 upstream regions or 100 downstream of a known TSS, then it is considered as true positive. On the other hand, if a predicted promoter region occurs within the coding regions of the gene, then it is regarded as a false positive prediction. A true positive region may have more than one prediction, in that case, the nearest prediction (or lsp) to the TSS has been considered. PromPredict achieves ~80% recall and ~37% precision in whole genome prediction (Table 4), which is comparable to its performance in prokaryotes^17^. Furthermore it has been shown in rice and Arabidopsis that a chunk of predictions in intronic regions given by PromPredict are potential *cis-*regulators^28^ but in *S*. *cerevisiae*, the intronic regions represent only a total of 3.8% of the ORFs. Predictions of various types of transcripts belonging to protein coding (ORF) as well as non-coding RNAs (CUTs, SUTs and other RNAs such as rRNA, tRNA and SnoRNA) has been considered for checking the performance of the algorithm. Precision, recall and F-score values shown in Table 4 suggest that PromPredict can annotate putative promoters of different classes of transcripts. It should be noted that PromPredict identifies the promoters associated with SUTs and CUTs which are related to pervasive transcription^12^.

**Table 4:** Whole genome promoter prediction in *S*. *cerevisiae* using “PromPredict”.

	Number of sequences	Transcript median length	TP	FP	TP promoters	Precision	Recall	F-score
ORF	4912	1548	5317	9072	3934	37.0	80.1	50.6
Cuts	501	428	540	242	404	69.1	80.6	74.4
Suts	729	964	727	704	552	50.8	75.7	60.8
Other	300	1272	296	447	223	39.8	74.3	51.9
All	6442	1436	6880	10465	5113	39.7	79.4	52.9
**TATA and TATA-less promoters**								
TATA	842	1384	978	1298	701	43.0	83.3	56.7
TATA-less	4070	1544	4206	7529	3139	35.8	77.1	48.9

Promoter prediction for 16 chromosomes for both forward and reverse strands has been carried out. −500 to +100 region relative to transcript start was chosen as a true positive region. The performance of PromPredict has been evaluated using the parameters precision, recall, and F-score. Precision is the ratio of number of true positives to the sum of true and false positive predictions, while recall is the ratio of the numbers of promoters with an identified true positive gene to the total number of promoters. TATA-containing and TATA-less gene promoters are defined based on the criterion of presence of TATA-box in −150 to −1 region relative to TSS^22^. Recall values for prediction of different transcripts belonging to ORF, non-protein coding CUTs, SUTs and other RNA classes (tRNA, rRNA, and SnoRNA) suggest that PromPredict is a good predictor for yeast promoter sequences. The algorithm performs better for TATA-containing gene promoters as compared to TATA-less promoters.

In yeasts, gene promoters can be classified into TATA-box containing and TATA- less promoters based on the presence of TATAWAWR consensus motif ^33^ in the vicinity of TSS. In *S*. *cerevisiae* ~17% of the gene promoters belong to TATA- containing class. The two categories of genes vary in their biological functions and gene expression. Further, the TATA-containing promoters are shown to be flexible while TATA-less promoters are rigid in different species of yeast^34^. In *S*. *cerevisiae*, TATA-containing promoters are less stable, more bendable and slightly curved compared to TATA-less promoters^22^. PromPredict has been tested on the promoters of two classes of genes. Recall and precision values are ~83% and ~43% for TATA-containing and ~77% and ~36% for TATA-less genes respectively (Table 4). Better performance of PromPredict for TATA-containing gene promoters compared to TATA-less promoters can be attributed to the lower stability of promoters^22^ as well as smaller median length of ORFs in the former class^35^. The analysis has also been extended for genes with variable expression.

**Figure 6:**
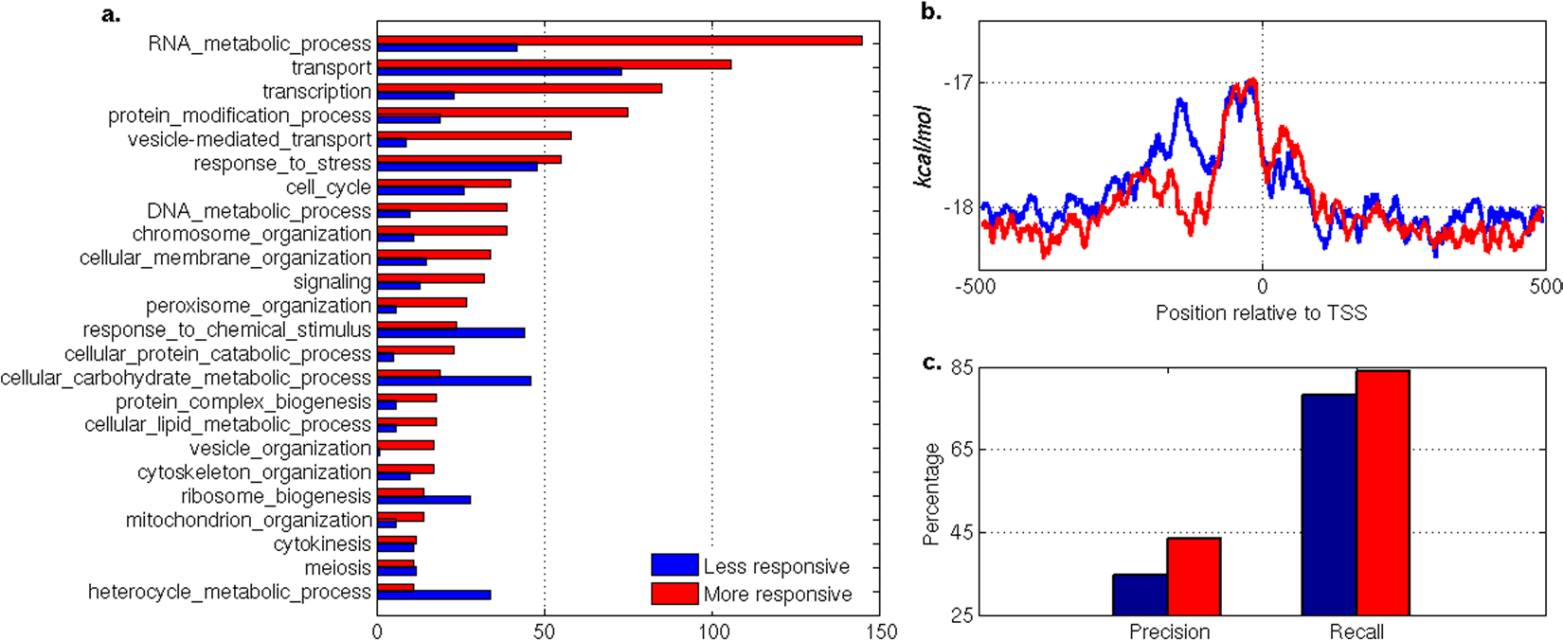
Performance of PromPredict in less responsive and more responsive genes. Top and bottom ten percentile list of gene (or) promoter sequences considered for this analysis. The dataset contains 459 genes in each category. (**a**) The frequency of genes in each class of biological process has been plotted to compare the two classes of genes. The less responsive genes are overrepresented in few biological processes compared to more responsive genes. The genes categorized in the gene ontology GO SLIM categories were retrieved from Saccharomyces genome database (http://www.yeastgenome.org/). (**b**) AFE profiles plotted using 15 nt window shows the differences in the distribution of low stability regions for two classes of gene promoters. (**c**) Precision and recall values shown as bar plot suggest that PromPredict comparatively a better predictor for more responsive genes. The −500 to +100 region relative to TSS is considered as true positive region.

Gene expression variability, which is essential for phenotypic variability, can be measured in the contexts of the environment, evolution, etc. Seven measures of variability, stochastic noise, responsiveness, stress response, *trans* variability, mutational variance, inter-strain variance and expression divergence have been used in an earlier study^36^. The performance of PromPredict was compared with high and low expression variability classes in all above mentioned measures (Supplementary Table 1). Genes with high or low expression variability are associated with specific biological functions. To define two categories, first, we have sorted the genes, based on their corresponding expression values and created ten bins, each containing 10% of the gene dataset. Top and bottom percentile bins are regarded as low and high expression categories. Precision, recall and F-score values for the seven measures suggest that less and more responsive genes exhibit major differences (Fig. 6c). In our earlier study, we have shown that gene responsiveness is intimately linked to DNA structural properties of promoters, nucleosome occupancy and promoter architecture^20^. Figure 6a shows an example of difference in the association of less responsive and more responsive genes with their biological function. The gene ontology (biological process) analysis shows that high responsive genes are linked to heterocyclic metabolic process, carbohydrate metabolism and response to chemical stimulus while being less responsive class associated with processes such as RNA metabolism, transcription, protein modification and transport etc. Distinct differences are also observed in free energy profiles of less responsive and more responsive genes. Less responsive genes have broader low stability region with two split peaks while more responsive ones have a comparatively narrow peak (Fig. 6b, additional file). More responsive genes (F-score = 57%) are better predicted by PromPredict compared to less responsive genes (F-score = 48%). The whole genome predictions suggest that PromPredict can be applied to yeast promoters and can be helpful to differentiate various classes of promoters.

## Conclusions

*In silico* promoter characterization is a great tool for identification of transcription initiation events, prediction of DNA-Transcription factor binding events and can help experimental molecular biologists to perform low-cost screening. Promoter prediction using in-house software PromPredict in the promoter sequences of six eukaryotic systems *S*. *cerevisiae*, *C*. *elegans*, *D*. *melanogaster*, zebrafish, mouse and human with transcription start site (TSS) data along with 48 eukaryotic systems with translation start site (TLS) data reveals that differential stability is a good criterion for promoter prediction. Whole genome prediction in *S*. *cerevisiae* using PromPredict suggests that it can be applied to yeast genomes, and its performance is observed to be different in distinct classes of genes such as TATA-containing and TATA-less, and variably expressed genes. Also, promoter prediction in 1001 nt long sequences flanking the TLS of 48 eukaryotes suggests that PromPredict is a good predictor of promoter regions in eukaryotes. Overall the recall values indicate that PromPredict can be applied for predicting promoter regions in different domains of life, even though it was initially designed for prokaryotes. PromPredict can be implemented without any specified cut-off to any genome, which makes it a very useful tool for global promoter annotation. The software is available as standalone as well as online version (http://nucleix.mbu.iisc.ac.in/prompredict/prompredict.html). Implementation of various sliding window sizes can be done on user’s request.

## Materials and Methods

### Promoter sequence datasets

Genome sequences of 48 different eukaryotes representing various domains of life are used in current study. Genomic sequences −500 and +500 relative to translation start site (TLS) for different species of invertebrates (*Caenorhabditis elegans*, *C*. *brenneri*, *C*. *briggsae*, *C*. *remanei*, *C*. *japonica*, *Drosophila melanogaster*, *D*. *annanassae*, *D*. *erecta*, *D*. *grimshawi*, *D*. *mojavensis*, *D*. *persimilis*, *D*. *pseudoobscura*, *D*. *sechellia*, *D*. *virilis*, *D*. *yakuba*, *Anopheles gambiae*, *Apis mellifera*, lancelet, sea squirt, sea hare), and vertebrates (zebrafish, fugu, lamprey, medaka, stickleback, tetraodon, mouse, human, chicken, cow, elephant, pig, platypus and rat) have been extracted from UCSC table browser^37^. Datasets for fungi are retrieved from the *Saccharomyces* Genome Database (SGD) (http://www.yeastgenome.org/) for Saccharomyces species (*S*. *cerevisiae*, *S*. *castellii*, *S*. *bayanus*, *S*. *kluyveri*, *S*. *kudriazvevii*, *S*. *mikatae and S*. *paradoxus*) and from the *Candida* Genome Database (CGD) (http://www.candidagenome.org/) for Candida and closely related species (*C*. *albicans*, *C*. *dubliniensis*, *C*. *glabrata*, *C*. *lusitaniae*, *C*. *tropicalis*, *Debaryomyces hansenii* and *Lodderomyces elongisporus*). Total number of sequences and the whole genome GC percentages are listed in Table 1. Transcription start site (TSS) information of three vertebrates, zebrafish, mouse, and human was obtained from Database of Transcription Start Sites (DBTSS), database version 7.0^38^. TSS information of *C*. *elegans* and *D*. *melanogaster* was retrieved from modENCODE database (http://www.modencode.org/) and Graveley *et al*.^39^ transcriptome profiling studies respectively. In case of *S*. *cerevisiae* transcript positions with both ends mapped have been considered^12^. Transcript information of 4912 open reading frames (ORFs), 501 cryptic unstable transcripts (CUTs), 729 stable unannotated transcripts (SUTs) and 300 other transcripts (dubious ORF, tRNA, SnoRNA, rRNA, etc.) is used for whole genome promoter prediction. The TSS positions of each system are mapped to respective genomes and sequences upstream as well as downstream are extracted. Genomic sequence data is downloaded from SGD, Wormbase (https://www.wormbase.org/), Flybase (http://flybase.org/) and UCSC genome browser (https://genome.ucsc.edu/) for *S*. *cerevisiae*, *C*. *elegans*, *D*. *melanogaster* and vertebrates (zebrafish, mouse and human) respectively.

Promoter sequences used in this study are 1001 nt long, starting 500 nt upstream, extending up to 500 nt downstream of either TSS or translation start sites (TLS) and the position ‘0’ corresponds to TSS or TLS. 2001 nucleotide long sequences are also considered for mouse and human promoter with TSS data.

### TATA-containing and TATA-less promoters and gene expression variability data

Consensus sequence TATA[A/T]A[A/T][A/G]^33^ has been considered to extract TATA-containing and TATA-less gene promoters in *S*. *cerevisiae*. TATA-containing promoters are regarded as those sequences, which contain TATA-box within −150 to −1 region relative to TSS^22^. Gene expression data of *S*. *cerevisiae* for seven different gene expression variations namely stochastic noise, responsiveness, stress response, *trans* variability, mutational variance, inter-strain variance and expression divergence is taken from Choi and Kim 2009 study^36^. Top and bottom ten percentile expression variability values have been used to define genes with lowest and highest expression variability categories^20^.

### DNA duplex stability or Free energy calculation

DNA duplex stability is referred to the ability of DNA to open up or melt, depends on its hydrogen bonding and base pair stacking. The dinucleotide step energy values corresponding to the 16 dinucleotide steps are taken from melting studies on 108 oligonucleotides^40^. Free energy calculations are computed using 15 nucleotide sliding window^27^.

### Promoter Prediction

Promoter prediction analysis in 48 different eukaryotic systems is carried out using in-house algorithm “PromPredict”^17,27^. The promoter predictions in mouse and human are also achieved using other structure-based algorithm EP3^16^.

### PromPredict

PromPredict discriminates between promoter and non-promoter sequences by using the most informative energy related feature, DNA duplex stability. The algorithm considers the Gibbs free energy change (average free energy) of a DNA stretch, which is computed as a sum of the constituent of dinucleotides over two 100 nt (or 50 nt) segments separated by 50 nt window, and the difference of energy values between two segments are evaluated and GC percentage based cut-off values are used to call putative promoter regions^17^. The scoring function D(n) is used to look for differences in free energy of the two neighbouring regions with respect to every nucleotide position ‘n’ (equation 1). The average energy is assigned to the centre position ‘n + 50’ corresponding to each 100 nt window.

1 $$D(n)=E1(n)-E2(n)$$

2 $$E1(n+50)=\frac{{\sum }_{n}^{n+100}{\rm{\Delta }}G^\circ }{100}$$

3 $$E2(n+50)=\frac{{\sum }_{n+150}^{n+250}\,{\rm{\Delta }}G^\circ }{100}$$

The functions E1(n + 50) and E2(n + 50) correspond to the mean free energy for 100 nt segments starting from nucleotide position ‘n’ and ‘n + 150’, respectively (equations 2 and 3). D(n) is the difference between E1 and E2. A stretch of DNA sequence is assigned as a promoter only if its average free energy (E1) and the difference in free energy (D(n)) as compared to its neighbouring downstream region are greater than the chosen cut-off values for the respective %GC range, as defined in the TSS- TLS based cut-off values^17^. ‘PromPredict’ outputs predicted promoter region, start and end of the predicted promoter region along with the least stable position (lsp) in the predicted promoter region. In the current study, the ‘lsp’ of a predicted region has been chosen as a measure for defining true and false predictions.

The algorithm is scalable and flexible, allowing the setting of sliding window sizes, such as [50 + 25 + 50], [100 + 50 + 100] or [200 + 50 + 200]. The default [100 + 50 + 100] window size restriction has been implemented based on previous analyses^27,41^. However, a statistical analysis on efficiency of “PromPredict” algorithm for various window sizes has been presented as boxplot (Supplementary Figure 7). The recall, precision and F-score were calculated for *S*. *cerevisiae* chromosomes (16) for both forward and reverse strands separately. It should be noted that the default window is best suited for both quantitative (recall) and qualitative (precision) analysis as revealed by the F-scores. Notably, with the bigger sliding window (such as 200), the computational time and ambiguity in assigning promoter location increases.

### Easy Promoter Prediction Program (EP3)

The EP3 program implements a base-stacking property to demarcate promoter regions from other genomic regions. For a given sequence of DNA, it computes inverted base-stacking energy values over a predefined window size in non-overlapping fashion and calls a DNA fragment as a promoter when the feature value crosses the genome specific threshold score^10,16^. EP3 uses two empirically determined parameters: length of the window and deviation of feature value from genomic average. Window size, 400 has been defined as optimal, and threshold-values of base-stacking are predetermined for genome size. The EP3 program is available at the site: http://bioinformatics.psb.ugent.be/webtools/ep3.

### Evaluation of performance

To assess the performance of PromPredict or EP3, evaluating parameters, recall, precision and F-score (harmonic mean of recall and precision) have been used (equations 4–6). These measures for the predictions are calculated using the following formulae:

4 $$Recall=\frac{No.\,\,of\,gene\,promoters\,with\,an\,identified\,True\,Positive\,genes}{Total\,number\,of\,genepromoters}$$

5 $$Precision=\frac{True\,\,Positives}{True\,Positives+False\,Positives}$$

6 $$F \mbox{-} Score=\frac{2\ast Precision\ast Recall}{Precision+Recall}$$

Several true positive regions have been used for eukaryotes for assessing the performance of promoter predictors such as [−1000, +1000] nucleotide^42^, [−500, +500] nucleotide and [−500, +100] nucleotide^28^. In this study, the [−500, +100] nucleotide region is considered for evaluating the performance of PromPredict for both TSS (six systems) and TLS (48 systems) datasets. To compare PromPredict with EP3, the true positive (TP) region is chosen as [−1000, +1000] as the latter program gives a prediction in 400 nt non-overlapping windows. False positive (FP) prediction is regarded as the prediction outside the TP region, but for whole genome predictions, only the predictions that lie in the coding regions are chosen, and other predictions are omitted. The closest prediction for a TP gene is considered as a strong signal, which might correspond to the core promoter region^28^.

## Electronic supplementary material

Supplementary information

Supplementary dataset
