# Supplementary material for: Identification of putative promoters in 48 eukaryotic genomes on the basis of DNA free energy
Source: Sci Rep. 2018 Mar 14;8:4520. doi: 10.1038/s41598-018-22129-8 (PMC5852095; doi:10.1038/s41598-018-22129-8)
Supplement: Supplementary file 1 — Supplementary information [file 41598_2018_22129_MOESM1_ESM.docx]

**Identification of putative promoters in 48 eukaryotic genomes on the basis of DNA free energy**

**[Venkata Rajesh Yella^1, 2^, Aditya Kumar^1, 3^ and Manju Bansal^1, *^**]

^1^Molecular Biophysics Unit, Indian Institute of Science, Bangalore, Karnataka, India 560012

^2^Department of Biotechnology, Koneru Lakshmaiah Education Foundation, Vaddeswaram, Guntur, Andhra Pradesh, India522502

^3^Present address: Department of Molecular Biology and Biotechnology, Tezpur University, Tezpur, Napaam, Assam, India 784028

^*^Corresponding author: ***Manju Bansal***

Email: [mb@iisc.ac.in](mailto:mb@iisc.ac.in)

**
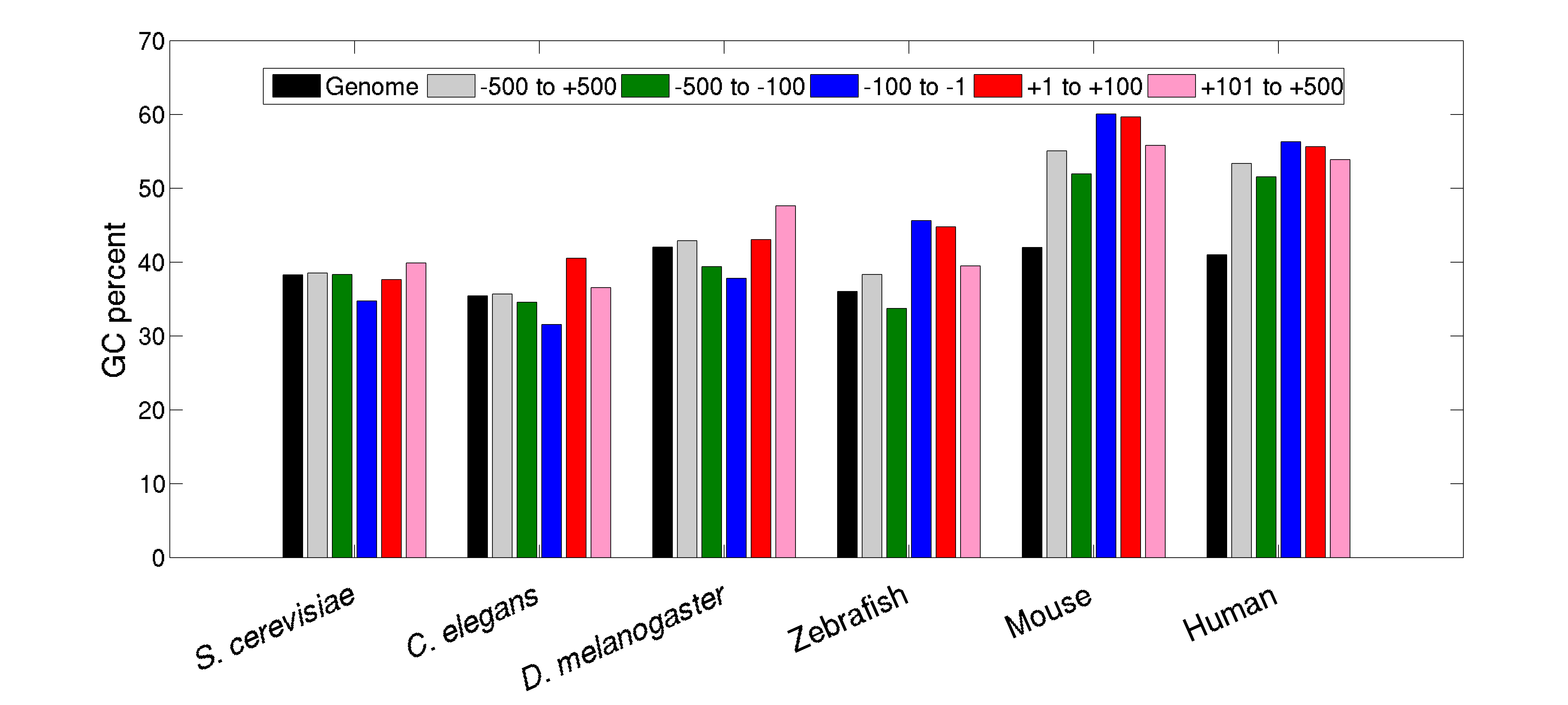
**

**Supplementary Figure 1: GC percent distribution in different regions relative to transcription start sites (TSSs)**. The mean GC percent in the various promoter regions are calculated and shown as bars. For comparison, whole genome GC percentages are also displayed. In lower eukaryotes (*S. cerevisiae*, *C. elegans,* and *D. melanogaster*), the GC percentage of core promoter region (-100 to -1 region) is lower compared to whole genome as well as other promoter regions (-500 to -100, +1 to +100 and +100 to +500) while in mouse and human, the results are quite opposite.

**
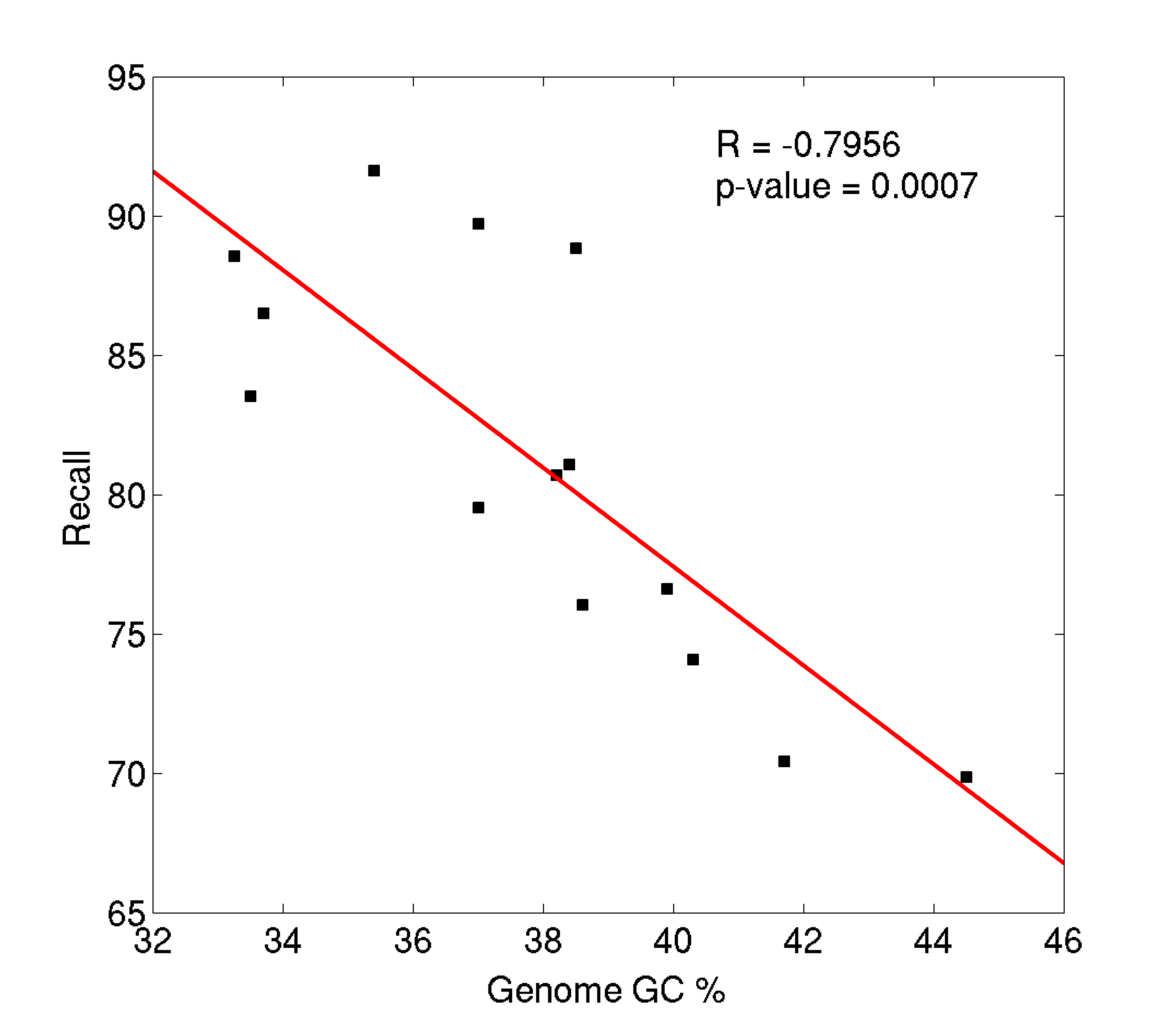
**

**Supplementary Figure 2: Correlation between Recall and Genome GC percentage in 14 yeast species**. Pearson’s correlation coefficient (R) has been calculated. R and P-values suggest that recall is negatively correlated with genome GC content in yeast species.

**
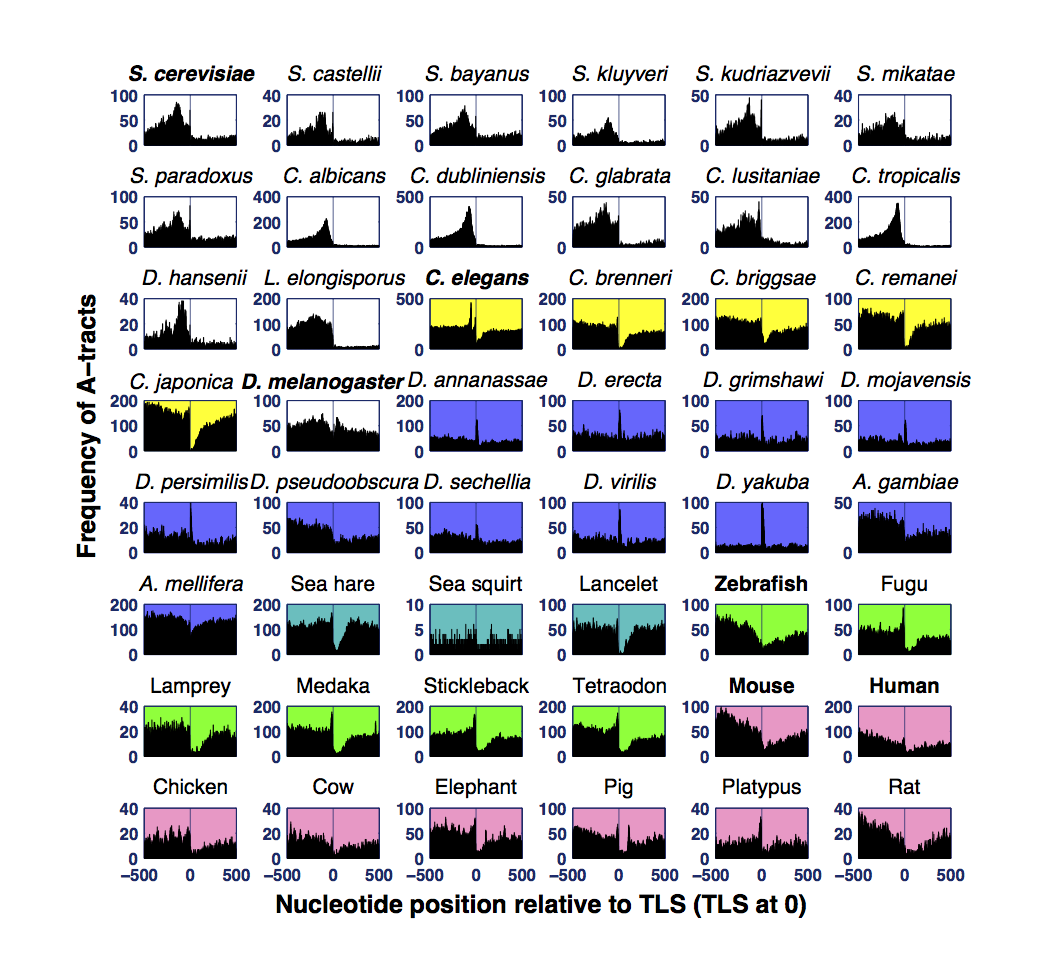
Supplementary Figure 3: Positional distribution of A-tracts in promoter regions of different eukaryotes**. A7 or T7 motifs are searched in -500 to +500 region relative TLS and summed at each nucleotide position and plotted. A-tracts are comparatively enriched in upstream regions of TLS in majority of eukaryotic systems. Color codes for the background of subplots (white for fungi, yellow for worms, purple for flys, cyan for marine invertebrates, green for fishes and pink for mammals and bird) have been used to differentiate between different domains of life. Fungal species, *S. cerevisiae*, *S. castellii*, *S. bayanus*, *S. kluyveri*, *S. kudriazvevii*, *S. mikatae*, *S. paradoxus*, *C. albicans*, *C. dubliniensis*, *C. glabrata*, *C. lusitaniae*, *C. tropicalis*, *D. hansenii* and *L. elongisporus* show high prevalence of A7 orT7 just upstream of TLS.

**
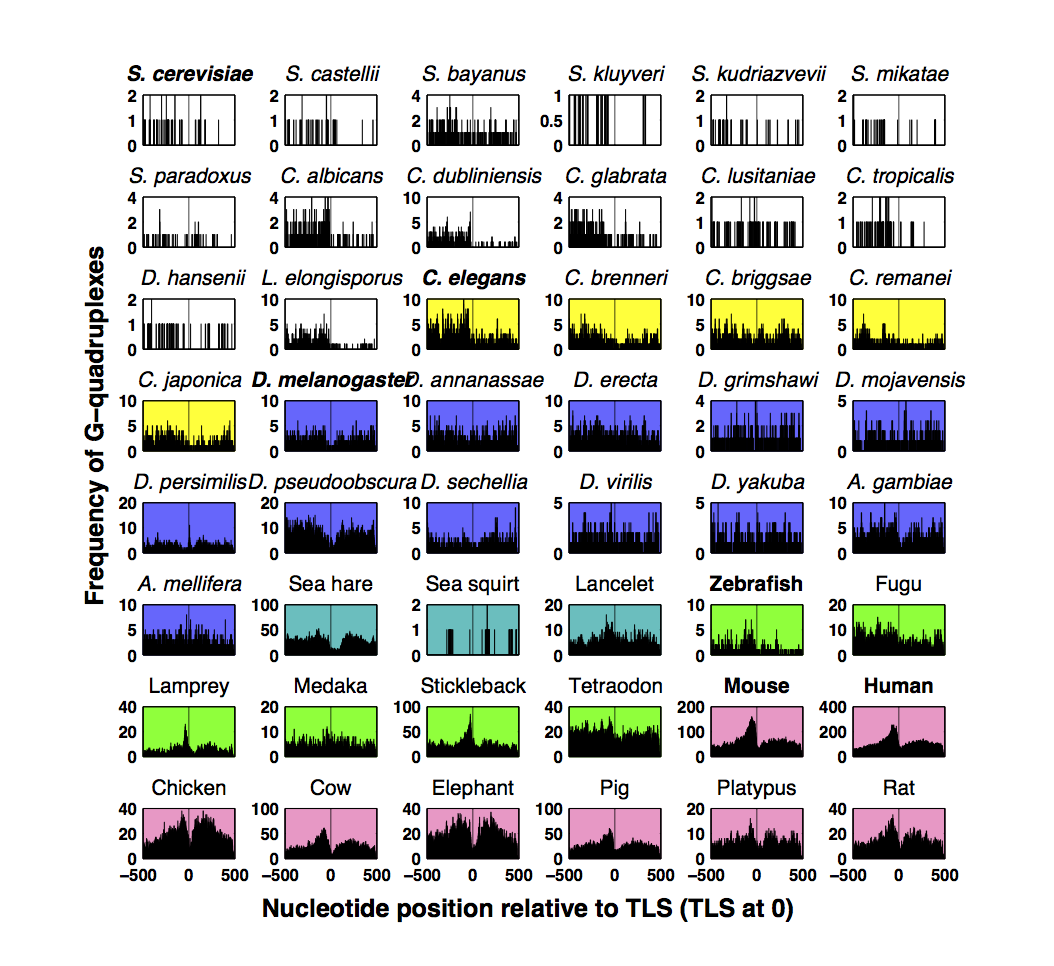
Supplementary Figure 4: Positional distribution of potential G-quadruplexes in promoter regions of different eukaryotes**. The regular expression G_3−5_N_1−7_G_3−5_N_1−7_G_3−5_N_1−7_G_3−5_ orC_3−5_N_1−7_C_3−5_N_1−7_C_3−5_N_1−7_C_3−5_ is searched in -500 to +500 region relative TLS and summed at each nucleotide position and plotted. Mammalian promoter regions are significantly enriched with G-quadruplexes in the vicinity of TSS.

**
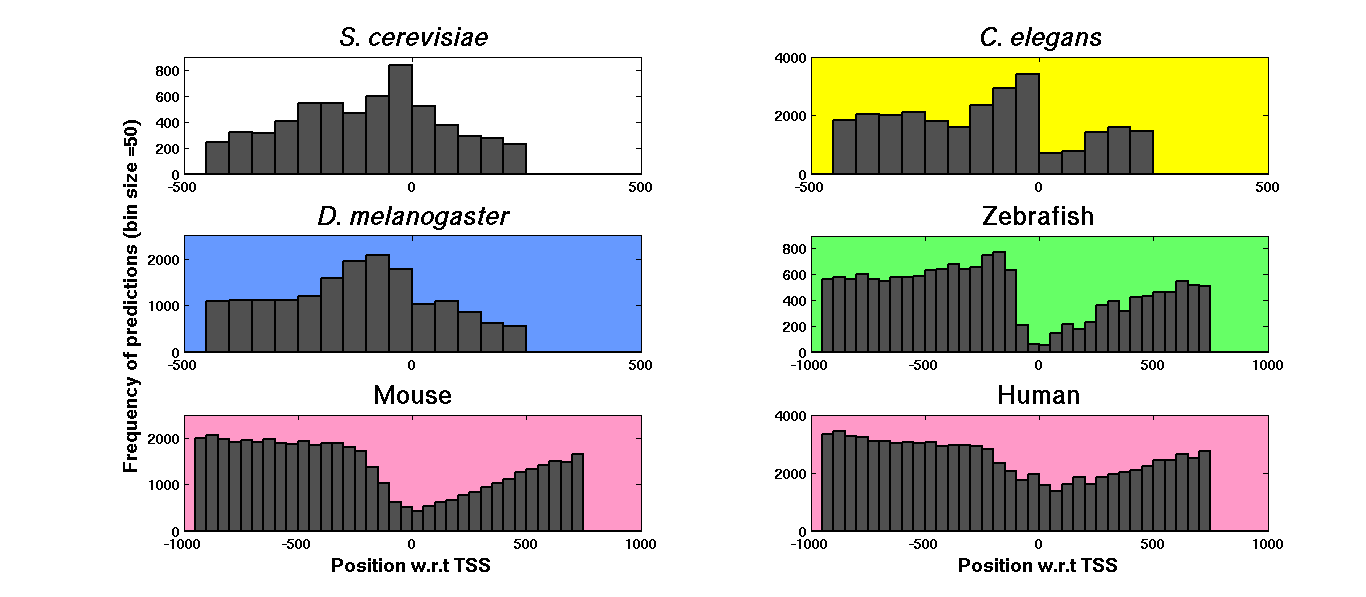
**

**Supplementary Figure 5: Positional distribution of predictions in promoter regions of different eukaryotes with TSS data.** The plot shows the distance of predictions from TSS, in 50nt bins, for *S. cerevisiae*, *C. elegans*, *D. melanogaster*, zebrafish, mouse, and human. The promoter regions of *S. cerevisiae*, *C. elegans,* and *D. melanogaster* show maximum predictions close to the TSS (in upstream region) while in mammals predictions are clustered in both upstream and downstream regions.


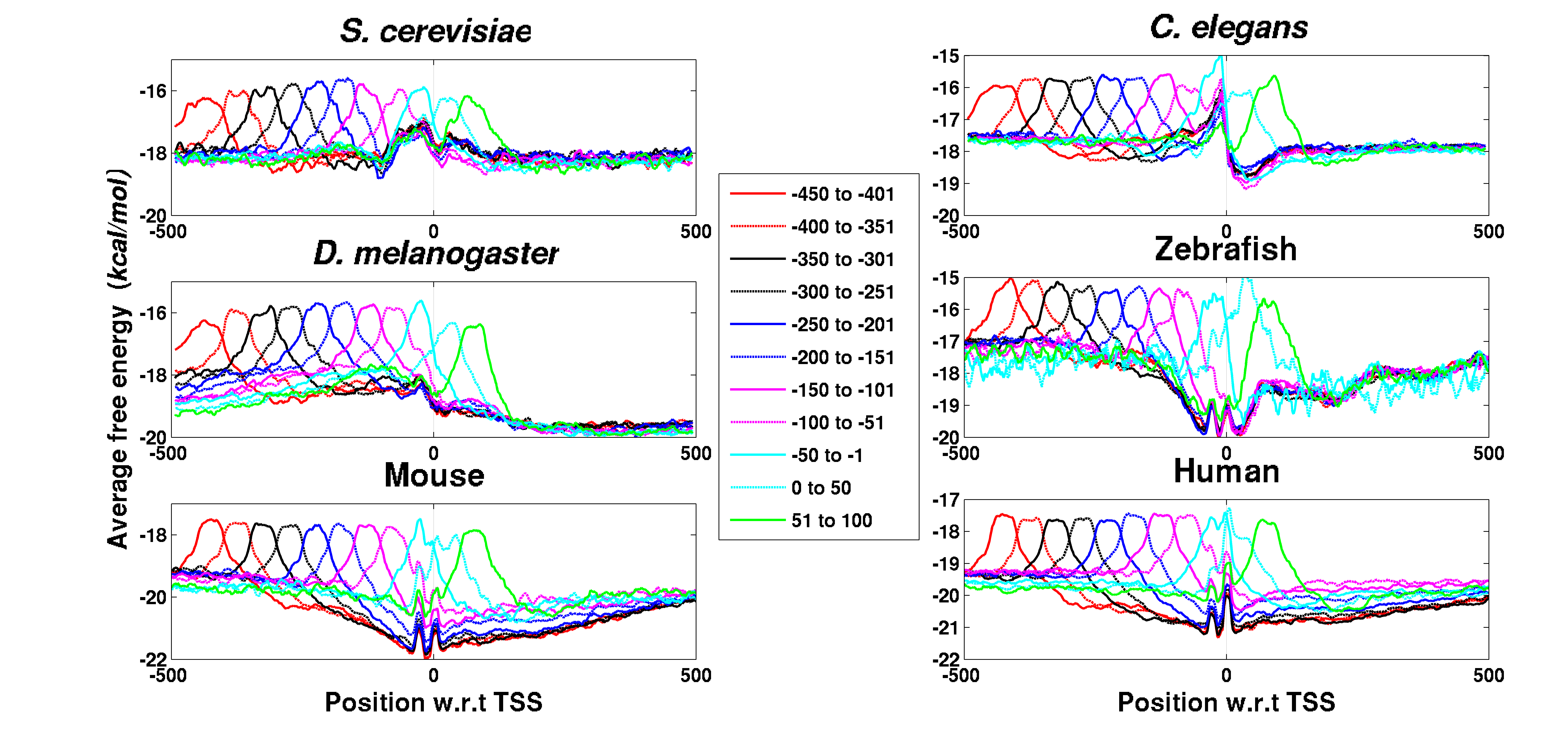


**Supplementary Figure 6: AFE profiles of the promoter sequences belonging to different TP classes.** The promoter sequences with predictions in the TP region (-500 to +100) are categorized into different TP classes based on 50nt bins of predictions shown in Supplementary figure 6, and their DNA duplex stability profiles are plotted.


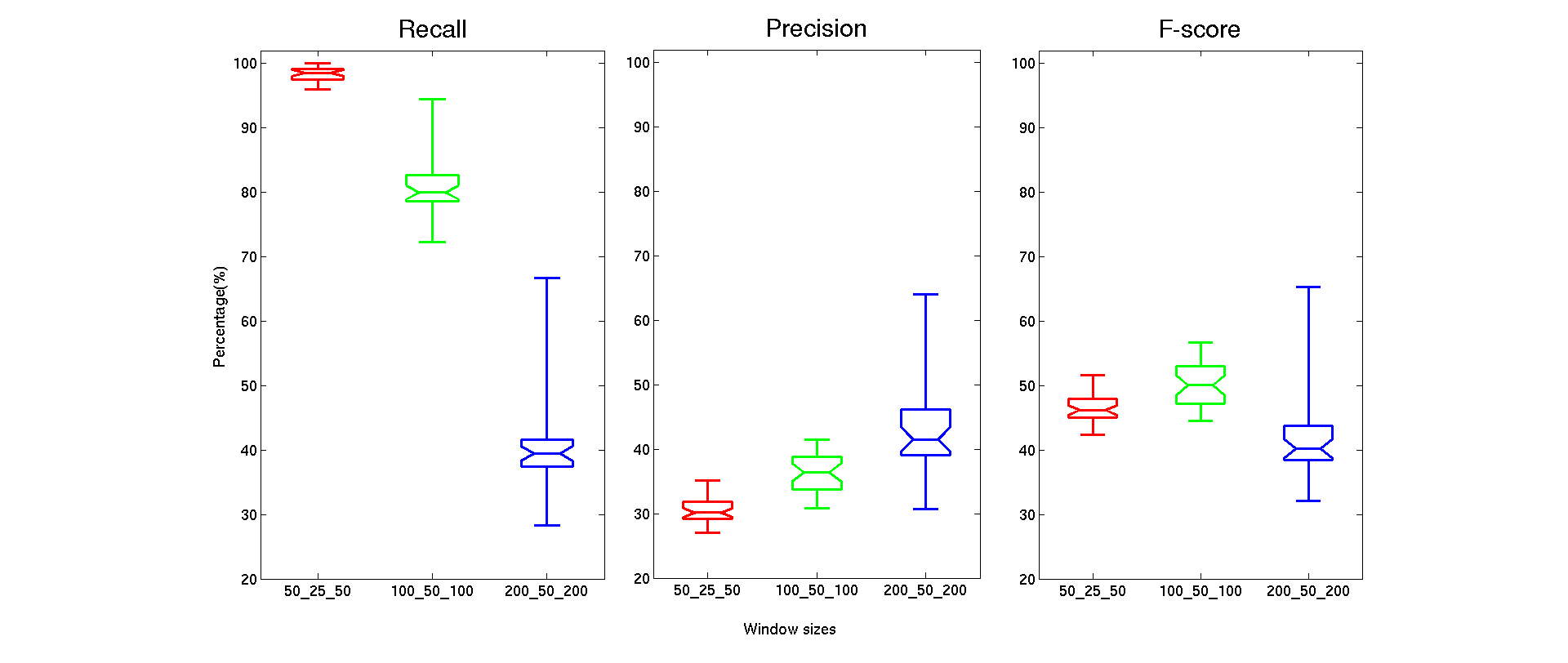


**Supplementary Figure 7: Comparison of performance of PromPredict with different sliding window sizes in *S. cerevisiae* whole genome:** The red, green and blue colored boxes (from left to right) in the plot show the efficiency of PromPredict with window sizes, 50-25-50, 100-50-100 (default window size implemented in the original algorithm) and 200-50-200 respectively. F-score values suggest that 100-50-100 window is the best for promoter prediction.

| **Gene expression variability** | | | | | | |
| --- | --- | --- | --- | --- | --- | --- |
|  | **High** | | | **Low** | | |
|  | **Precision** | **Recall** | **F-score** | **Precision** | **Recall** | **F-score** |
| Stochastic noise | 37.5 | 82.2 | 51.5 | 37.7 | 82.7 | 51.8 |
| Responsiveness | 43.5 | 83.9 | 57.3 | 34.6 | 78.2 | 48.0 |
| Stress response | 36.5 | 79.2 | 50.0 | 38.2 | 82.6 | 52.3 |
| *trans* variability | 36.7 | 80.7 | 50.4 | 38.0 | 82.2 | 52.0 |
| Mutational variance | 36.0 | 78.5 | 49.3 | 37.1 | 82.9 | 51.3 |
| Inter-strain variance | 36.3 | 80.3 | 50.0 | 35.7 | 79.7 | 49.3 |
| Expression divergence | 38.4 | 84.8 | 52.8 | 37.7 | 78.5 | 50.9 |

**Supplementary Table 1: Performance of PromPredict in high and low gene expression variability classes.** Gene expression data of *S. cerevisiae* for seven different gene expression variations namely stochastic noise, responsiveness, stress response, *trans* variability, mutational variance, inter-strain variance and expression divergence are taken from a recently compiled study (Choi and Kim, 2009). High and low expression variability categories have been defined as mentioned in (Yella and Bansal, 2013). The -500 to +100 fragment relative to TSS is chosen as true positive region for predictions. PromPredict performs differently for genes showing high and low expression variability, with maximum differences being observed for responsiveness.
